# Serum Lipidomic Profile Signature of Active Acromegaly and Relationships to Cardiovascular Disease

**DOI:** 10.3390/ijms27021082

**Published:** 2026-01-21

**Authors:** Oana Stănoiu-Pînzariu, Thalijn L. C. Wolters, Carmen Socaciu, Cristina Alina Silaghi, Ana Valea, Ioana Popa-Ilie, Georgeta Hazi, Andreea Iulia Socaciu, Romana Teodora Netea-Maier, Carmen Emanuela Georgescu

**Affiliations:** 1 5th Department of Medical Sciences, Department of Endocrinology, Iuliu Haţieganu University of Medicine and Pharmacy, 400349 Cluj-Napoca, Romania; oana_pinzariu@yahoo.com (O.S.-P.); alinasilaghi@yahoo.com (C.A.S.); ana74us@yahoo.com (A.V.); ioanamanaila@yahoo.com (I.P.-I.); 2 Endocrinology Clinic, Cluj County Emergency Clinical Hospital, 400349 Cluj-Napoca, Romania; georgetahazi@gmail.com; 3 Department of Internal Medicine, Division of Endocrinology, Radboud University Medical Center, 6525 Nijmegen, The Netherlands; thalijn.wolters@radboudumc.nl (T.L.C.W.); romana.netea-maier@radboudumc.nl (R.T.N.-M.); 4 RTD Center of Applied Biotechnology BIODIATECH, SC Proplanta, 400478 Cluj-Napoca, Romania; csocaciu@proplanta.ro; 5 Department of Occupational Health, Iuliu Haţieganu University of Medicine and Pharmacy, 400347 Cluj-Napoca, Romania; andreea.socaciu@umfcluj.ro

**Keywords:** lipidomics, GH-secreting pituitary adenoma, acromegaly, mass spectrometry, high-performance liquid chromatography

## Abstract

Acromegaly is a rare endocrine disease characterized by multiple metabolic abnormalities and high cardiovascular risk. This cross-sectional study evaluated the lipidomic serum profile of 109 participants (59 acromegaly patients versus 50 healthy controls) via high-performance liquid chromatography combined with mass spectrometry (HPLC-MS). The lipidomic profile that differentiated acromegaly from controls included sphingomyelins (SMs), glycerophospholipids, glycerolipids, ceramides, fatty acids, wax esters (WEs), carnitines, and sterol (ST) lipids. SM 34:0;O2 and phosphorylcholine best distinguished acromegaly patients from controls (VIP > 2.49). SM 34:0;O2 levels were significantly elevated in treatment-naïve versus uncontrolled patients (*p* < 0.0001). Furthermore, SM 34:0;O2 positively correlated with random GH and IGF-1. Lack of therapy predicted SM 34:0;O2 serum titers in acromegaly. Profound alterations of glycerophospholipids and sphingolipids were detected in acromegaly patients with cardiovascular complications. ST 24:1;O3, ceramide (Cer) 38:0;O4, and WE 34:1 were significantly increased in both hypertensive acromegaly patients and those with heart failure in comparison to patients without cardiovascular impairment. In conclusion, SM 34:0;O2 and phosphorylcholine emerged as potential lipidomic biomarkers in acromegaly. Moreover, SM 34:0;O2 potentially reflects disease severity. Identifying lipidomic profile alterations in acromegaly patients with cardiac involvement may provide a basis for further insights into the cardiovascular pathogenesis of the disease.

## 1. Introduction

Acromegaly is a rare endocrine disease typically caused by a growth hormone (GH)-secreting pituitary adenoma [1]. Even with diagnostic and therapeutic advances, patients with acromegaly still have increased cardiovascular morbidity and mortality rates [2]. In spite of the relatively uniform clinical manifestations of acromegaly, there is a considerable heterogeneity in the patients’ responses to therapy [3]. Lipidomics could offer a powerful tool to elucidate this heterogeneity, providing a deeper understanding of the molecular mechanisms underlying acromegaly and its associated complications.

The investigation of lipidomic signatures in acromegaly is still at an early stage [4,5,6,7]. Using liquid chromatography combined with mass spectrometry (LC-MS), Fellinger et al. [4] observed significantly reduced concentrations of sphingomyelin (SM) d36:0, butyrylcarnitine, hexanoylcarnitine, as well as phosphatidylcholine (PC) species (e.g., PC 36:5, PC O-38:6, PC O-42:5, and PC O-40:7) in the plasma of 15 patients with active acromegaly [4]. Through ^31^P/^1^H-magnetic resonance spectroscopy (MRS) of the liver, the authors demonstrated a decrease in hepatocellular lipids and enhanced hepatic mitochondrial activity, indirectly reflected by reduced levels of carnitine species in acromegaly patients [4].

In a metabolomic research conducted on 68 patients with various pituitary adenomas (including 8 patients with acromegaly), Banerjee et al. detected significant serum lipids profile alterations using LC-MS [5], as patients with acromegaly harboured elevated serum levels of E-parinaric acid—a polyunsaturated fatty acid (FA), and C16 phytosphingosine, alongside with decreased glycerophospholipids (e.g., lysophosphatidylcholine (LPC) (18:3(9Z,12Z,15Z)), LPC (20:5(5Z,8Z,11Z,14Z,17Z), 1-heptadecanoyl-sn-glycero-3-phosphoethanolamine, 1-linoleoyl-sn-glycero-3-phosphoethanolamine, 1-Oleoyl-2-hydroxy-sn-glycero-3-phosphoethanolamine, and 1-linoleoyl-2-hydroxy-sn-glycero-3-PC). Also, increased levels of 3-hydroxydodecanoylcarnitine and decreased levels of stearoylcarnitine and cis-4-Decenoyl carnitine were noticed in the serum of patients with acromegaly [5].

A notable study by Wang et al. [6] comprehensively characterized the plasma lipidomic profile of 80 treatment-naïve acromegaly patients using high-performance LC-MS (HPLC-MS). All patients underwent metabolic evaluation, including basal glucose, lipid profile, lipoprotein(a), apolipoproteins A1 and B, and were also assessed by echocardiography. A total of 42 compounds were identified in the plasma samples of acromegaly patients, most of which were glycerophospholipids and fatty acids (FAs). The most representative lipidomic pathways identified by Wang et al. [6] in acromegaly included sphingolipid metabolism, glycerophospholipid metabolism, and linoleic acid metabolism. In addition, the authors identified positive correlations of LPC 16:0 with left ventricular (LV) ejection fraction and shortening fraction, and between phosphatidylethanolamine (PE) 22:6/16:0 and LV mass [6]. The results of this study represent a first step towards understanding the role of lipidomic alterations in the cardiovascular complications of acromegaly [6].

To date, other lipidomic studies on cardiovascular comorbidities in acromegaly patients have not yet been reported in the literature.

In a meta-analysis of two representative cohorts of 2642 participants from the Framingham Heart Study and 3134 from the Study of Health in Pomerania, it was shown that the plasma ceramide (Cer) 24:0/Cer 16:0 ratio was inversely associated with the risk of heart failure and coronary heart disease [8,9]. In addition, in a study involving more than 4000 participants, elevated plasma levels of Cer 16:0 and SM 16:0 were associated with an increased risk of heart failure [10]. Using LC-MS, Wittenbecher et al. [11] validated Cer 16:0 and PC 32:0 as predictive factors for heart failure. Moreover, the latter author identified lipid clusters associated with heart failure, comprising sphingolipids (e.g., Cer 16:0, SM 18:0 and 18:1), phospholipids, plasmalogens, FAs, diacylglycerols (DGs), and triacylglycerols [12]. Zhao et al. identified seven compounds via LC-MS, six of which were lipids (e.g., PE species (16:0p/20:3, 16:0e/20:4, 18:0p/18:2, and 18:1p/18:2), (O-acyI)-1-hydroxy FA (36:3), and cafestol). 

In the PREMID trial, which evaluated 983 participants, glycerolipids and PEs were associated with the risk of coronary heart disease [13]. The most representative lipid species identified in calcific aortic valve stenosis was sterol (ST) ester 27:1/17:1 [9,14]. In aortic valve tissue with severe stenosis, elevated concentrations of LPC species (16:0 and 18:0) and omega-6 polyunsaturated FAs were detected [9,15]. In addition, significant perturbations in glycerophospholipid metabolism, primarily of PC, LPC, and PE species, have been observed in patients with dilated cardiomyopathy [16].

The present study focused on serum lipidomic profiling in acromegaly patients, seeking to identify differentially abundant lipid species in acromegaly versus healthy subjects. Secondly, we aimed to assess the lipid species associated with cardiovascular comorbidities (e.g., hypertension, heart failure, and acromegalic cardiomyopathy) in acromegaly patients, in addition to their potential predictive value.

Although our study may appear similar to Wang’s research [6], serum lipidomic analysis could reveal lipid species that differ from those identified in plasma. Furthermore, we aimed to identify potential correlations between the most representative lipid species detected in the acromegaly group and (1) the main disease activity parameters, such as IGF-1 and random GH, as well as (2) other metabolic parameters, including basal glucose, total cholesterol (TC), LDL-cholesterol (LDL-C), HDL-cholesterol (HDL-C), and triglycerides (TG). Additionally, we sought to establish lipidomic links between acromegaly and its cardiovascular complications.

Thus, the detection of lipidomic signatures of acromegaly and the quantification of potential lipidomic biomarkers could (1) facilitate early diagnosis of the disease and thereby improve prognosis, (2) enable cardiovascular risk stratification, and (3) lead to a better understanding of the underlying molecular pathogenic mechanisms, ultimately guiding the development of personalized therapies for affected patients.

## 2. Results

### 2.1. Patient Data

**Figure 1:**
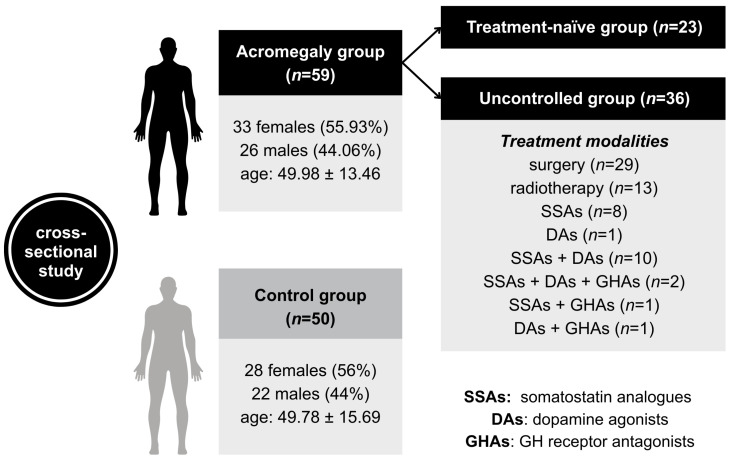
The flowchart of study groups. Age is expressed in years as mean ± standard deviation (SD). The acromegaly group included 23 treatment-naïve patients and 36 patients who had undergone various types of treatment. The transsphenoidal approach was used for pituitary surgery in 29 patients. Seven patients underwent conventional radiotherapy, while six received stereotactic radiotherapy. The figure depicts in detail the numbers of patients on either drug monotherapy or combination treatment. SSAs: somatostatin analogues; DAs: dopamine agonists; GHAs: GH receptor antagonists.

The acromegaly group included 59 patients with active acromegaly (33 females) aged 49.9 ± 13.4 (mean ± standard deviation (SD)) years with a BMI of 30.2 ± 5.5 (mean ± SD) kg/m^2^. The control group included 50 healthy subjects (28 females) aged 49.7 ± 15.6 (mean ± SD) years, with a BMI of 27.7 ± 4.8 (mean ± SD) kg/m^2^. Twenty-three of the total number of acromegaly patients were treatment-naïve, whereas thirty-six were treated, though uncontrolled (i.e., IGF-1 levels remained elevated, at 1.3 times the upper limit of normal (ULN)). Twenty-nine of the thirty-six treated patients underwent surgery, thirteen received radiotherapy, and twenty-three received medication. Drug treatment included somatostatin analogues (SSAs, *n* = 21), GH receptor antagonists (GHAs, *n* = 4), and dopamine agonists (DAs, *n* = 14). Combinations of drugs were administered in 14 subjects (Figure 1).

The clinical and biochemical features of patients with acromegaly are detailed in Table 1. The treatment-naïve group had higher IGF-1 and random GH levels than the uncontrolled group (*p* < 0.0001 and *p* = 0.0006, respectively). The average time between diagnosis of acromegaly and study enrolment was 9.3 months in the treatment-naïve group versus 86.6 months in the uncontrolled group (*p* < 0.0001), the latter treated according to standard national therapeutic protocols. In addition, concentrations of basal glucose, homeostatic model assessment for insulin resistance (HOMA-IR), and LDL-C were higher in treatment-naïve patients than in the uncontrolled patients (*p* < 0.05 for all three metabolic parameters). Central hypothyroidism was more common in the uncontrolled group than in the treatment-naïve group (*p* < 0.05).

### 2.2. Serum Lipidome of Acromegaly

**Figure 2:**
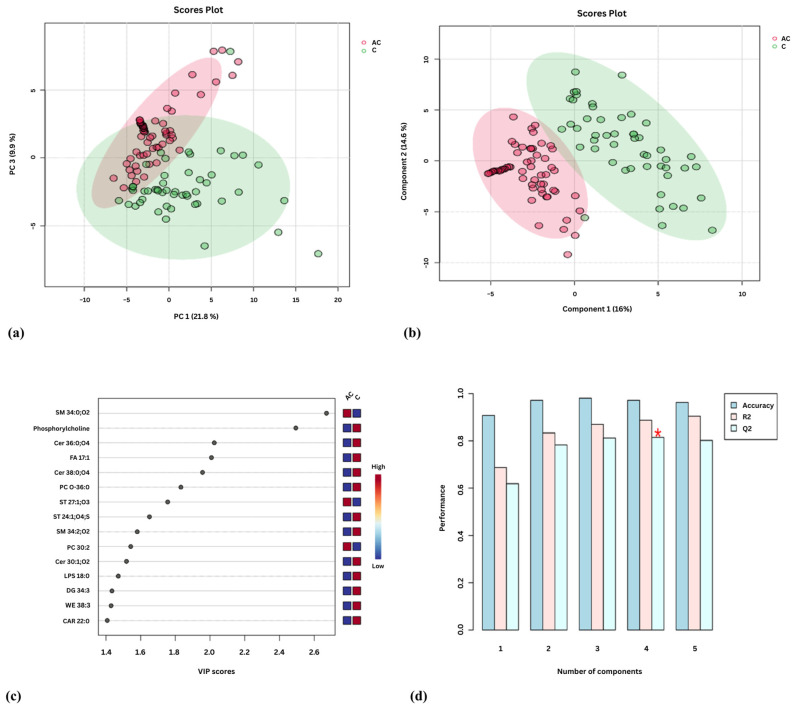
Serum lipidomic analysis in acromegaly: (**a**) Principal Component Analysis (PCA) score plot; (**b**) Partial least squares discriminant analysis (PLS-DA) score plot. Each point on the plot represents an individual sample, and acromegaly samples are in red, while control samples are in green; (**c**) Variable Importance in Projection (VIP) score derived from PLS-DA; (**d**) PLS-DA cross-validation data (leave-one-out cross-validation [LOOCV]). The red star indicates the best classifier.

We performed lipidomic profiling of sera from acromegaly patients alongside healthy subjects (controls). Through Principal component analysis (PCA), we observed only slight separation between the two cohorts: acromegaly vs. controls (Figure 2a). Subsequently, partial least squares discriminant analysis (PLS-DA) showed a clear distinction between the two cohorts (Figure 2b,c). Cross-validation of the PLS-DA model revealed cumulative values of R2 of 88% and Q2 of 81.5% (Figure 2d). Ninety-one lipid species predominated in serum samples (>80%) and were considered eligible for statistical analysis. The top 15 of these lipid species with the variable importance in projection (VIP) score above 1.4 are presented (Figure 2c and Table 2). Three lipid species increased and 12 decreased in acromegaly compared to healthy controls (Figure 2c and Table 2). These compounds belong to the following lipid classes: (1) sphingomyelins (SMs), (2) glycerophospholipids, (3) ceramides, (4) FAs, (5) glycerolipids, (6) carnitines, (7) wax esters, and (8) ST lipids (Figure 2c and Table 2).

Of all the lipid species identified, SM 34:0;O2 most accurately discriminated acromegaly from controls (VIP = 2.67). SM 34:2;O2 was another lipid species that discriminated between the two study groups (VIP = 1.58). In patients with acromegaly, SM 34:0;O2 levels were significantly elevated in serum, whereas SM 34:2;O2 levels were markedly decreased (Figure 2c and Table 2).

The two most discriminating glycerophospholipids in the acromegaly group were phosphorylcholine and PC O-36:0 (VIP = 2.49 and VIP = 1.83, respectively). Other compounds belonging to the class of glycerophospholipids included PC 30:2 and lysophosphatidylserine (LPS) 18:0. With the exception of PC 30:2, most of the lipid species mentioned above exhibited decreased concentrations in acromegaly patients (Figure 2c and Table 2).

Concerning the three species of ceramides (Cer 30:1;O2, Cer 36:0;O4, and Cer 38:0;O4), all were decreased in acromegaly (Figure 2c and Table 2).

Sterols (ST) 27:1;O3 and 24:1;O4;S, belonging to the class of ST lipids, discriminated patients with acromegaly from the control group (VIP = 1.76 and VIP = 1.65). ST 27:1;O3 was increased and ST 24:1;O4;S was decreased in patients with acromegaly (Figure 2c and Table 2).

In addition, DG 34:3, belonging to the glycerolipids class, carnitine (CAR) 22:0, FA 17:1, and wax ester (WE) 38:3 were present at reduced serum levels in the acromegaly group compared with the control group (Figure 2c and Table 2).

The list of 62 molecules identified and their species label according to Lipidmaps is presented in Table S1. Table 2 includes the top lipid species with VIP scores above 1.4 and their fold change (FC), log2 (FC), and *p*-values, as well as the significance of their variation.

### 2.3. Relationships Between Lipid Species and IGF-1 and GH

The correlations of lipid species with IGF-1 and random GH are detailed in Table 3. Seven lipid metabolites correlated with both variables mentioned above. Thus, a positive correlation between SM 34:0;O2 and random GH (r = 0.434, *p* = 0.000) and IGF-1 (r = 0.270, *p* = 0.038) was noticed. Also, SM 34:0;O2 was negatively related to the time interval from diagnosis to study enrolment (r = −0.399, *p* = 0.001). Additionally, ceramide phosphate (CerP) 32:1;O2, DG 34:1, PE 28:1, PC 28:0, phosphatidic acid (PA) 32:5, and LPC 20:3 negatively correlated with IGF-1 and random GH, as shown in Table 3.

### 2.4. Relationships Between Lipid Species and Classic Metabolic Parameters in Acromegaly

The basal glucose was positively correlated with SM 34:0;O2 (r = 0.28, *p* = 0.035), PC 30:2 (r = 0.287, *p* = 0.03), and monogalactosyl monoacylglycerol (MGMG) 18:2 (r = 0.381, *p* = 0.003) (Table 4). TG was positively correlated with Cer 36:0;O4 (r = 0.326, *p* = 0.012) (Table 4). TC was positively correlated with Cer 38:0;O4 (r = 0.297, *p* = 0.023) and ST 24:1;O3 (r = 0.266, *p* = 0.043). A positive correlation was observed between LDL-C and LPS O-20:0;O (r = 0.33, *p* = 0.012). HDL-C was positively correlated with LPS O-18:0 (r = 0.358, *p* = 0.006) and negatively correlated with WE 34:1 (r = −0.287, *p* = 0.032), WE 38:7 (r = −0.293, *p* = 0.028), DG 32:1 (r = −0.357, *p* = 0.006), and CE 15:0 (r = −0.285, *p* = 0.033) (Table 4).

### 2.5. Lipidomic Profile That Discriminates the Treatment-Naïve Acromegaly Group from the Uncontrolled Group

**Figure 3:**
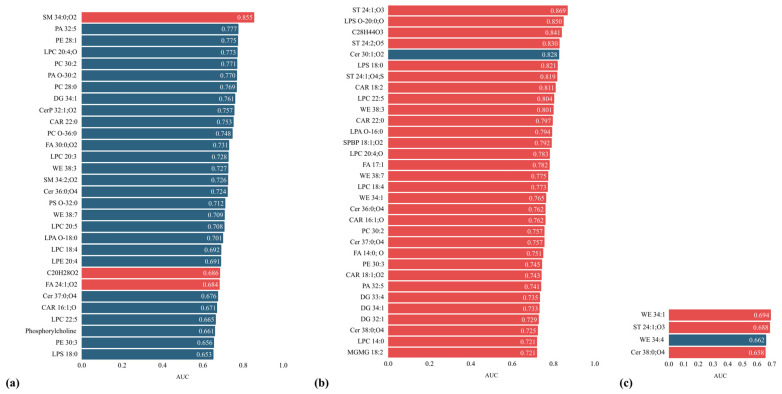
The profile of lipid species identified in (**a**) treatment-naïve acromegaly patients; and in patients bearing acromegaly and cardiovascular complications: (**b**) heart failure and (**c**) hypertension. Red corresponds to an increasing trend, while blue corresponds to a decreasing trend. AUC: Area Under the Curve.

Thirty lipid species discriminated the treatment-naïve from the uncontrolled group (adjusted *p* < 0.05). These were represented by (1) SM species (SM 34:0;O2, SM 34:2;O2), (2) sphingolipids (CerP 32:1;O2, Cer 36:0;O4, and Cer 37:0;O4), (3) phosphorylcholine, (4) glycerophospholipids (PA O-30:2, PA 32:5, PE 28:1, PE 30:3, PC 28:0, PC 30:2, PC O-36:0, phosphatidylserine (PS) O-32:0, lysophosphatidic acid (LPA) O-18:0, lysophosphatidylethanolamine (LPE) 20:4, LPC 18:4, LPC 20:3, LPC 20:4;O, LPC 20:5, LPC 22:5, and LPS 18:0), (5) DG 34:1, (6) carnitines (CAR 16:1;O and CAR 22:0), (7) FAs (FA 24:1;O2 and FA 30:0;O2), (8) C20H28O2, and (9) WEs (WE 38:3 and WE 38:7) (Figure 3a).

**Figure 4:**
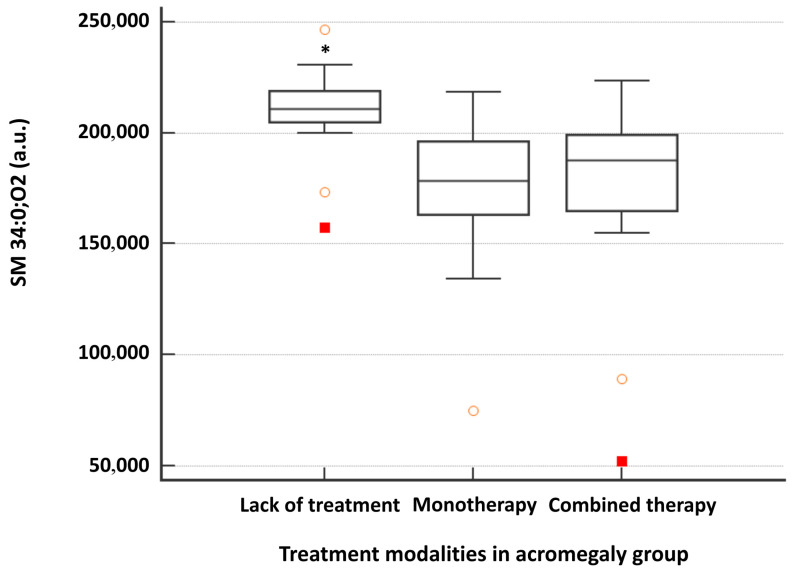
Serum level of SM 34:0;O2 in the acromegaly group according to the type of therapy administered. Monotherapy consisted of surgery, pharmacological therapy, or radiotherapy. Combined therapy consisted of at least two therapeutic modalities, namely surgery, pharmacological therapy, and radiotherapy. * *p*-value was <0.05 between the untreated group (treatment-naïve) (*n* = 23) and both monotherapy (*n* = 14) and combined therapy (*n* = 22) groups (one-way ANOVA—Scheffé test).

Of these, SM 34:0;O2 was the lipid species with the highest discriminatory power among the study groups (*p* < 0.0001, AUC = 0.855) (Figure 3a). One-way analysis of variance (ANOVA) was used to assess potential predictors of SM 34:0;O2 serum titres in patients with acromegaly. The absence of treatment was identified as a prognostic predictor for SM 34:0;O2 levels in acromegaly patients (F = 8.03, *p* = 0.001), with significantly higher titres observed in untreated patients compared with those receiving treatment, either monotherapy or combined therapy (Figure 4).

Except SM 34:0;O2, C20H28O2, and FA 24:1;O2, the rest of the lipid species detected in treatment-naïve acromegaly patients showed lower titres than in treated patients with acromegaly, as depicted in Figure 3a.

### 2.6. Discriminatory Lipidomic Profile of Cardiovascular Complications in Acromegaly Patients

#### 2.6.1. Discriminatory Lipidomic Profile of Heart Failure in Acromegaly Patients

Thirty-two lipid species discriminated acromegaly patients with heart failure from those with normal cardiac function (adjusted *p* < 0.05) (Figure 3b). These included (1) carnitine species (CAR 16:1;O, CAR 18:1;O2, CAR 18:2, and CAR 22:0), (2) sphingolipids (Cer 30:1;O2, Cer 36:0;O4, Cer 37:0;O4, Cer 38:0;O4, and sphingosine-1-phosphate (SPBP) 18:1;O2), (3) glycerolipids (MGMG 18:2, DG 32:1, DG 33:4, and DG 34:1), (3) ST lipids (ST 24:1;O3, ST 24:1;O4;S, and ST 24:2;O5), (4) WEs (WE 34:1, WE 38:3, and WE 38:7), (5) FAs (FA 14:0;O and FA 17:1), (6) glycerophospholipids (PA 32:5, PC 30:2, PE 30:3, LPA O-16:0, LPC 14:0, LPC 18:4, LPC 20:4;O, LPC 22:5, LPS 18:0, and LPS O-20:0;O), and (7) C28H44O3 (Figure 3b).

All these lipid species were elevated, except for Cer 30:1;O2, which had a lower concentration in the presence of heart failure. Ten lipid species out of the 32 showed an AUC value greater than 0.8. These were ST lipids (ST 24:1;O3, ST 24:1;O4;S, and ST 24:2;O5), LPC 22:5, LPS 18:0, LPS O-20:0;O, C28H44O3, Cer 30:1;O2, CAR 18:2, and WE 38:3 (Figure 3b).

All 32 lipid species were individually introduced as independent variables in a logistic regression analysis to identify potential predictors for heart failure in acromegaly. LPA O-16:0 (*p* = 0.016), LPS O-20:0;O (*p* = 0.004), and ST 24:1;O3 (*p* = 0.004) were retained, but with odds ratio (OR) = 1, 95% confidence interval (CI) ranging between 1–1.0001.

#### 2.6.2. Lipidomic Profile of Patients with Acromegaly and Hypertension

Four lipid species discriminated acromegaly patients with hypertension from those with normal blood pressure (adjusted *p* < 0.05) (Figure 3c). These were WE 34:1, WE 34:4, ST 24:1;O3, and Cer 38:0;O4. Of these, WE 34:1 had the highest discriminatory power (*p* = 0.01, AUC = 0.694) (Figure 3c). Except for WE 34:4, all lipid species were elevated in hypertensive acromegaly patients compared to normotensive acromegaly patients (Figure 3c).

All four lipid species were individually introduced as independent variables in a logistic regression analysis to identify potential predictors for hypertension in acromegaly. Thus, WE 34:4 (*p* = 0.023) and Cer 38:0;O4 (*p* = 0.044) were retained, but with OR = 0.99, 95% CI 0.99–1 for WE 34:4 and OR = 1, 95% CI 1–1.0002 for Cer 38:0;O4, respectively.

#### 2.6.3. Lipidomic Profile of Acromegaly Patients with Cardiomyopathy

No lipid species differed significantly between acromegaly patients with and without acromegalic cardiomyopathy.

## 3. Discussion

The serum lipidomic fingerprint obtained in acromegaly patients via untargeted HPLC-MS revealed compounds from several lipid classes, including SMs, glycerophospholipids, ceramides, FAs, glycerolipids, carnitines, WEs, and ST lipids.

Our findings align with previous research. The alterations in lipid metabolism in our acromegaly cohort were consistent with the results of studies conducted by Fellinger et al., Banerjee et al., and Wang et al. [4,5,6], which primarily identified changes in glycerophospholipids and sphingolipids. As a novelty, our research additionally assessed the relationships between the lipidomic signature and disease activity biomarkers, such as random GH and IGF-1. Furthermore, it was demonstrated here that therapeutic intervention might significantly impact the lipidomic signature in acromegaly, despite a lack of complete disease control. Finally, we specifically sought to identify potential links between the lipidomic panel and disease-related cardiovascular comorbidities.

In our research, SM 34:0;O2 and phosphorylcholine most effectively discriminated the acromegaly group from healthy controls. Sphingomyelins, integral components of the cell membranes, partnering with cholesterol to organize membrane microdomains, comprise ceramide and phosphorylcholine in their structure [17]. Phosphorylcholine plays a crucial role in phospholipid synthesis, while sphingomyelins are involved in maintaining membrane integrity and function [18]. SM 34:0;O2 was elevated in treatment-naïve acromegaly patients, showing a positive correlation with random GH levels and a negative correlation with the time interval from diagnosis to study enrolment. Aside from that, SM 34:0;O2 was weakly associated with IGF-1 and serum glucose concentration. Moreover, the absence of any therapeutic intervention predicted higher titres of SM 34:0;O2 in patients with acromegaly (F = 8.03, *p* = 0.001), suggesting that SM 34:0;O2 may reflect disease severity. The observed increase in saturated species such as SM 34:0;O2 alongside the decrease of polyunsaturated species, e.g., SM 34:2;O2 in the serum of acromegaly patients underpins complex lipid metabolism disturbances induced by GH/IGF-1 excess. This hypothesis could be explained by the existence of insulin resistance and enhanced lipolysis in acromegaly, which alter the availability of FAs for ceramide synthesis within sphingolipid metabolism [19,20].

Besides SM 34:0;O2, the majority of lipid species detected in treatment-naïve acromegaly patients and exhibiting AUC values above 0.75 were glycerophospholipids (e.g., PA 32:5, PE 28:1, LPC 20:4;O, PC 30:2, PA O-30:2, and PC 28:0). Several glycerophospholipid compounds have previously been described in pituitary adenomas [21,22]. Thus, ^31^P-MRS revealed decreased levels of phosphatidylserine (PS) and increased concentrations of PC and phosphatidylinositol in brain tissues obtained from patients with pituitary adenoma [21].

Wang M et al. [6] reported a positive correlation between LPC 16:0 and LV fractional shortening and ejection fraction, as well as between PE 22:6/16:0 and LV mass in patients with a diagnosis of acromegaly, thereby suggesting an involvement of glycerophospholipids in cardiovascular impairment. Our results are in agreement with the findings of Wang et al., by revealing pronounced alterations in glycerophospholipid metabolism, especially in acromegaly patients with heart failure (e.g., increased levels of PA 32:5, PC 30:2, PE 30:3, LPA O-16:0, LPC 14:0, LPC 18:4, LPC 20:4;O, LPC 22:5, LPS 18:0, and LPS O-20:0;O). Among these metabolites, the most representative compounds, achieving an AUC value above 0.8, were LPC 22:5, LPS 18:0, and LPS O-20:0;O.

Other notable compounds detected at elevated titres in acromegaly patients with heart failure included ST lipids (e.g., ST 24:1;O3, ST 24:1;O4;S, and ST 24:2;O5), 1,25-dihydroxyvitaminD2, WE 38:3, and CAR 18:2 (AUC > 0.8). ST 24:1;O3, WE 34:1, and Cer 38:0;O4 were lipid species distinctly recognized in both hypertensive acromegaly patients and those with heart failure in comparison to patients without cardiovascular impairment.

As in Fellinger’s study [4], in the research we performed, low ceramide titers differentiated patients with acromegaly from controls [4]. Sphingolipids have been intensively investigated in cardiovascular disease through targeted lipidomic studies, and Cer 16:0 has been established as a predictive factor for heart failure [8,9,10,11] and sudden cardiac death [23]. Elevated ceramide levels have been observed in hypertensive rats and in humans, in plasma and aortic tissue (for the animal model), contributing to the heightened vascular tone characteristic of hypertension [24]. Furthermore, Cantalupo et al. demonstrated the role of ceramides in blood pressure homeostasis in an animal model [25], suggesting that Cer 38:0;O4, observed in increased titres in our acromegaly cohort with hypertension and heart failure, could play a key role in the cardiovascular pathogenesis of the disease. In patients with acute myocardial infarction, 12 ceramide species were found to predict 12-month major adverse cardiovascular events, Cer(d18:1/24:1(15Z)), Cer(d18:1/22:1), and dihydroceramide (d18:0/16:0) being the most representative [26].

In the Cardiovascular Health Study, elevated sphingomyelin species containing palmitic acid were linked to heart failure [10] and sudden cardiac death [23]. A plausible mechanism involved is by promoting inflammation, a process that may connect acromegaly with cardiovascular disease [27].

Diacylglycerol species (e.g., DG 32:1, DG 33:4, and DG 34:1) were detected at elevated titres in our acromegaly patients with heart failure. A potential role of these molecules in cardiovascular pathogenesis may be implicated, as Razquin et al. conducted a large case-cohort study of 983 participants and concluded that, alongside PE species, glycerolipids, including DGs, were directly associated with an increased risk of cardiovascular disease [13].

Disturbances of the FAs profile were noted in our acromegaly cohort, as in the research of Banerjee et al. [5] and Wang et al. [6]. The alterations likely result from the lipolytic effect of GH [14]. Species of WEs were detected in higher amounts in patients with acromegaly, both with heart failure and with hypertension, although these compounds were not previously characterized to play a crucial role in classical lipid metabolism or in cardiovascular pathogenesis, primarily serving a protective role for the organism against dehydration and pathogens [28].

The sterol ST 24:1;O3 was identified at elevated concentrations in acromegaly patients with both hypertension and heart failure. Similarly, ST 27:1/17:1 was observed at elevated titres in the plasma of patients with aortic stenosis, a condition that may contribute to the development of heart failure or, indirectly, to hypertension [14].

Furthermore, we observed a positive correlation between basal glucose and MGMG 18:2, and between HDL-C and LPS O-18:0, in addition to a negative correlation between HDL-C and DG 32:1, CE 15:0, WE 34:1, and WE 38:7 in patients with acromegaly. These correlations have not been previously reported in the literature. Considering that DGs are involved in inducing insulin resistance [29], the negative association between DG 32:1 and HDL-C may be plausible, even if indirectly. Further studies will be needed to confirm these correlations and to elucidate potential underlying mechanisms. Additionally, TC showed positive correlations with Cer 38:0;O4 and ST 24:1;O3; LDL-C was positively correlated with LPS O-20:0;O, whereas TG showed a positive correlation with Cer 36:0;O4. The link between ceramide species, TC, LDL-C, and TG has been previously documented in the literature and may contribute to the enhanced atherosclerosis and cardiovascular risk [30,31].

Our research limitations include the cross-sectional design and the relatively small cohort size. Future prospective and large-scale studies are needed to clarify lipidomic dynamics in acromegaly and elucidate molecular mechanisms underlying acromegaly.

The results of our research could contribute to a better understanding of the disease pathogenesis and provide a starting point for the implementation of personalized medicine in acromegaly.

## 4. Materials and Methods

### 4.1. Study Cohorts

A cross-sectional study that enrolled 109 subjects (59 active acromegaly patients and 50 age and gender-matched healthy controls) was conducted in two European academic centres (Endocrinology Clinic of the Cluj County Emergency Clinical Hospital, Cluj-Napoca, Romania, and Radboud University Medical Center (Radboudumc), Nijmegen, The Netherlands). The study’s protocol was directed in accordance with the Declaration of Helsinki and approved by the academic Ethics Committees. Each participant gave informed consent before entering the study.

Active acromegaly was defined as IGF-1 levels above 1.3 × ULN accompanied by characteristic clinical features in treatment-naïve patients or as inadequate biochemical control in treated patients [32].

Subjects with acromegalic habitus associated with an intact somatotropic axis, immunosuppressive treatments, amino acid supplements, inadequately controlled diabetes mellitus (HbA1c > 10%), uncontrolled hypertension under antihypertensive treatment, and active malignancies were excluded from the study.

The time interval from diagnosis to study enrolment was defined as the time frame elapsed between the diagnosis of acromegaly and the moment of enrolment in the study. Central hypogonadism was established based on low oestrogen (in premenopausal women), low total testosterone (in men), and low or normal gonadotropin levels. The diagnosis of central hypothyroidism was based on low FT_4_ (<0.61 ng/dL) and low or normal TSH. Central hypocortisolism was established based on low cortisol (<5 µg/dL) and low or normal ACTH or insufficient cortisol stimulation (<20 µg/dL) after administration of 250 µg Synacthen or insulin tolerance test. All acromegaly patients with concomitant pituitary deficiencies were adequately managed with replacement therapy prior to study enrolment. The diagnosis of diabetes mellitus was based on a basal glucose level above 126 mg/dL or a glucose value above 200 mg/dL after two hours in an oral glucose tolerance test (OGTT). Prediabetes was defined as a basal glucose level between 100 and 126 mg/dL or a glucose value between 140 and 199 mg/dL after two hours in OGTT. Hypertension was confirmed by identifying systolic blood pressure above 140 mmHg and diastolic blood pressure above 90 mmHg or by taking antihypertensive drugs. Cardiomyopathy was established based on ventricular hypertrophy, diastolic malfunction, and progressive systolic impairment. The diagnosis of heart failure was made based on clinical manifestations described by the Framingham study [33] and left ventricular ejection fraction measured by echocardiography below 45%.

### 4.2. Anthropometric Measurements

All patients were clinically evaluated by measuring their blood pressure in the supine position, height, weight, hip, and waist circumference.

### 4.3. Assays

Prior to blood sample collection, all participants fasted for a minimum of 8 h, avoided unusual physical activity and psychological stress, and discontinued drugs and/or nutritional supplements (e.g., vitamins, amino acids) for at least 24 h. In premenopausal females, samples were collected between days 2 and 5 of the menstrual cycle.

Blood samples were collected by venipuncture into vacutainer tubes without anticoagulant from all participants, in the morning, before breakfast, and were let to clot at room temperature for 40 min. The sera obtained by centrifuging at 2000 rpm *g* for 10 min were divided into aliquots using 1-mL Eppendorf microtubes and stored at −80 °C.

Glucose, TC, LDL-C, and TG were measured spectrophotometrically (Mindray BC 6200, Mindray Bio-Medical Electronics Co., Ltd., Shenzhen, China). HDL-C was calculated after the Friedewald formula: HDL-C = TC − LDL-C − (TG/5). IGF-1 (Immulite 1000 system, Siemens Healthcare Diagnostics Products Limited, Llanberis, Gwynedd, LL554EL UK), GH (Cobas E411 Analyzer, Roche Diagnostics, Tokyo, Japan), prolactin, oestradiol (for women), testosterone (for men), FSH, LH, TSH, FT4, basal cortisol, and insulin (UniCel DxI 600 Access Immunoassay System, Beckman Coulter Inc., Brea, Cali-fornia, USA) were determined by respective chemiluminescence immunoassays. HOMA-IR was calculated after the following formula: HOMA-IR = fasting insulin (mIU/L) × glucose (mg/dL)/405 [34].

For the lipidomic analysis, 200 µL of serum was used, and 800 µL of methanol/acetonitrile 1:1 (*v*/*v*) solvent mixture was added to precipitate proteins.

The mixture was vortexed for 1 min, kept at 4 °C for 6 h, and then vortexed again for 1 min.

Subsequently, the vials were centrifuged for 5 min at 12,500 rpm, and the supernatant was recovered and filtered through nylon filters 0.2 µm. The lipidomic HPLC-MS/MS analysis was performed by using a C18 reverse-phase HPLC column (Acquity, UPLC C18 BEH, Dionex, Thermo Fischer Scientific, Waltham, MA, USA) mounted on a Thermo Scientific HPLC UltiMate 3000 system (Thermo Fisher Scientific, Waltham, MA, USA), equipped with a Dionex Ultimate quaternary pump delivery and coupled with electrospray ionization quadrupole time-of-flight mass spectrometry (ESI-QTOF-MS) mass spectrometer—Bruker Daltonics MaXis impact (Bruker GmbH, Bremen, Germany). The injected volume was 3 µL. The mobile phase was formed by mixing eluents A (water + 0.1% formic acid) and B (acetonitrile: methanol 1:1 + 0.1% formic acid). The gradient program was the following: 0 min 99% A, 1 min 70% A, 2 min 40% A, 6 min 20% A, 9–10 min 100% B (min 9–10), 15 min 99% A. The total run-to-run time was 15 min. The MS parameters were as follows: mass range: 50–1000 Da; the pressure of nebulizing gas: 2.8 bar; the temperature of drying gas: 300 °C; and the flow of drying gas: 12 L/min. The instrument was calibrated using sodium formate. The instrument control and data processing were performed using dedicated software provided by Bruker Daltonics (Compass Data Analysis 4.2, Hystar 3.2, and TofControl 3.2). Parallel quality control samples were generated by combining 0.2 mL of serum from each sample within each group. To ensure data repeatability, the quality control (QC) samples were run again after every ten samples. Each sample was analysed in duplicate.

### 4.4. Statistical Analysis

Data were analysed by Medcalc 19.3.1 software. Categorical variables were expressed as numbers (percentages). Continuous variables were expressed as mean (standard deviation). The Kolmogorov–Smirnov test was applied to verify the distribution of continuous variables. Pairwise statistical comparisons (acromegaly vs. controls) of continuous variables (lipid levels) were performed by the Mann–Whitney U test (for data distributions deviating from normal) or the T-test for independent samples (for normal data distributions). Fisher’s Exact test was employed to compare categorical variables between the two groups of samples. Correlation coefficients between the variables were calculated either by Pearson’s or Spearman’s tests, for normally or not normally distributed data, respectively. To identify potential prognostic factors in the study groups, either logistic regression or one-way ANOVA was conducted, depending on the type of dependent variables. To control the false discovery rate (type I errors) in lipidomic analysis, *p*-values were adjusted using the Benjamini–Hochberg procedure, as appropriate.

The data collected by measurements were processed using Compass Data Analysis 4.2. Initially, the individual recorded Total Ion Chromatograms (TIC) were transformed to Base Peak Chromatograms (BPC) and the compound spectra were recorded using the Find Molecular Feature (FMF) function, an algorithm of the Data Analysis software 4.2. The table released by Compass Data Analysis 4.2 contained the peak areas and intensities, the retention time, the signal/noise (S/N) ratio for each compound, and its *m*/*z* value. The number of detected lipid species varied between 600 and 800. Initially, a matrix for all samples was obtained and stored in an Excel file. A first filtration was used to remove the small signals with S/N values less than 10. After this, a second matrix containing peak intensities and *m*/*z* values was saved. A second filtration was used to remove the small intensities (less than 5000). Only the lipid species measured in over 80% of the samples were considered for data analysis. Thus, an aligned matrix was obtained using the NEAPOLIS web tool from bioinformatica.isa.cnr.it (http://bioinformatica.isa.cnr.it/NEAPOLIS/ accessed on 20 November 2025). After converting to a .csv file, the final data matrix was introduced into the MetaboAnalyst 4.0 web-based tool (https://www.metaboanalyst.ca accessed on 20 November 2025). After another round of successive alignment and normalization of the data matrix, Metaboanalyst was employed to compute FC, to make a Volcano plot (Figure S1), and to perform the multivariate analyses such as PCA and PLS-DA. The receiver operating characteristic (ROC) curves were used to evaluate the sensitivity/specificity of lipid species. Human Metabolome Database (https://hmdb.ca/ accessed on 20 November 2025) and LIPID MAPS^®^ Lipidomics Gateway (http://www.lipidmaps.org/ accessed on 20 November 2025) were used to identify the most representative lipid species.

## 5. Conclusions

Lipidomics provides a powerful approach for uncovering altered lipid metabolism in acromegaly. Phosphorylcholine and SM 34:0;O2 emerge as promising lipidomic biomarkers with the latter potentially reflecting disease severity. Identifying lipidomic profile alterations in acromegaly patients with cardiac involvement may provide a basis for further insights into the cardiovascular pathogenesis of the disease.

## Figures and Tables

**Table 1:** Clinical and biochemical features of acromegaly patients.

	All Acromegaly Patients (*n* = 59)	Treatment-Naïve Patients (*n* = 23)	Uncontrolled Patients (*n* = 36)	*p*-Value
Age (years)	49.98 (13.46)	51.82 (15.08)	48.80 (12.39)	0.293
Gender (female)	33 (55.93%)	11 (47.82%)	22 (61.11%)	0.421
BMI (kg/m^2^)	30.21 (5.59)	29.56 (6.35)	30.63 (5.09)	0.341
WHR	0.90 (0.04)	0.91 (0.6)	0.90 (0.04)	0.218
Systolic blood pressure (mmHg)	129.64 (18.49)	133.80 (20.70)	126.98 (16.69)	0.304
Diastolic blood pressure (mmHg)	82.88 (11.68)	85.82 (13.60)	81.01 (10.02)	0.103
IGF-1 (ng/mL)	432.75 (205.79)	566.60 (166.40)	347.23 (182.98)	**<0.0001**
Random GH (ng/mL)	7.35 (9.52)	11.54 (12.00)	4.43 (5.95)	**0.0006**
Time interval from diagnosis to study enrolment (months)	56.5 (92.8)	9.3 (45.03)	86.6 (103.1)	**<0.0001**
Basal glucose (mg/dL)	109.29 (34.01)	110.43 (18.47)	108.52 (41.64)	**0.047**
Insulin (µU/mL)	18.52 (11.65)	22.06 (11.97)	16.45 (11.10)	0.059
HOMA-IR	5.07 (3.81)	6.19 (3.67)	4.39 (3.79)	**0.027**
TC (mg/dL)	190.9 (38.28)	192.12 (42.91)	190.1 (35.55)	0.852
HDL-C (mg/dL)	52.23 (14.44)	55.05 (12.39)	50.53 (15.47)	0.234
LDL-C (mg/dL)	116.11 (33.03)	137.51 (54.37)	113.25 (31.38)	**0.026**
TG (mg/dL)	129.68 (57.73)	117.76 (61.80)	137.51 (54.37)	0.106
Pituitary insufficiency	23 (38.98%)	7 (30.43%)	16 (44.44%)	0.412
Central hypocortisolism	4 (6.77%)	0 (0%)	4 (11.11%)	0.148
Central hypothyroidism	15 (25.42%)	2 (8.69%)	13 (36.11%)	**0.030**
Central hypogonadism	16 (27.11%)	6 (26.08%)	10 (27.77%)	1.000
Diabetes mellitus	15 (25.42%)	8 (34.78%)	7 (19.44%)	0.228
Prediabetes	11 (18.64)	6 (26.08%)	5 (13.88%)	0.310
Hypertension	31 (52.54%)	14 (60.86%)	17 (47.22%)	0.423
Cardiomyopathy	9 (15.25%)	5 (21.73%)	4 (11.11%)	0.291
Heart Failure	8 (13.55%)	1 (4.34%)	7 (19.44%)	0.133

Continuous variables are expressed as mean (SD). Categorical variables are expressed as the number of patients with the particular trait (percentage of patients). *p*-values are from pairwise comparisons between the treatment-naïve and uncontrolled group. The statistically significant values (*p* < 0.05) are bolded.

**Table 2:** Lipidomic differences between acromegaly and control groups.

Species Label	VIP Score	FC	log2 (FC)	*p*-Value	Adjusted *p*-Value *	Significance AC Versus C
SM 34:0;O2	2.67	1.834	0.875	<0.0001	<0.0001	I
Phosphorylcholine	2.49	0.524	−0.933	<0.0001	<0.0001	D
Cer 36:0;O4	2.02	0.712	−0.490	<0.0001	<0.0001	D
FA 17:1	2.01	0.691	−0.534	<0.0001	<0.0001	D
Cer 38:0;O4	1.96	0.691	−0.533	<0.0001	<0.0001	D
PC O-36:0	1.83	0.686	−0.543	<0.0001	<0.0001	D
ST 27:1;O3	1.76	1.529	0.613	<0.0001	<0.0001	I
ST 24:1;O4;S	1.65	0.808	−0.308	<0.0001	<0.0001	D
SM 34:2;O2	1.58	0.476	−1.072	<0.0001	<0.0001	D
PC 30:2	1.54	1.403	0.488	<0.0001	<0.0001	I
Cer 30:1;O2	1.52	0.717	−0.480	<0.0001	<0.0001	D
LPS 18:0	1.47	0.732	−0.450	<0.0001	<0.0001	D
DG 34:3	1.43	0.809	−0.306	<0.0001	<0.0001	D
WE 38:3	1.43	0.824	−0.280	<0.0001	<0.0001	D
CAR 22:0	1.41	0.818	−0.290	<0.0001	<0.0001	D

Only the lipid species with *p* < 0.0001 and VIP score > 1.4 are listed. FC: fold change; D: decrease; I: increase. * The false discovery rate (FDR) adjustment using the Benjamini–Hochberg (BH) procedure was used to compute the adjusted *p*-value.

**Table 3:** Correlations of lipid species with IGF-1 and random GH.

Lipid Species Label	IGF-1 (ng/mL)		Random GH (ng/mL)	
	r	*p*	r	*p*
SM 34:0;O2	**0.270**	**0.038**	**0.434**	**0.000**
CerP 32:1;O2	**−0.305**	**0.018**	**−0.348**	**0.008**
DG 34:1	**−0.269**	**0.039**	**−0.314**	**0.018**
PE 28:1	**−0.343**	**0.007**	**−0.368**	**0.005**
PC 28:0	**−0.357**	**0.005**	**−0.363**	**0.006**
PA 32:5	**−0.310**	**0.017**	**−0.338**	**0.011**
LPC 20:3	**−0.245**	**0.006**	**−0.308**	**0.020**
ST 27:1;O3	0.166	0.207	**0.368**	**0.005**
SM 34:2;O2	−0.225	0.086	**−0.303**	**0.023**
LPE 20:4	−0.209	0.112	**−0.273**	**0.041**
ST 26:1;O4	0.247	0.059	**0.310**	**0.020**
C_30_H_46_O_3_	−0.037	0.779	**−0.300**	**0.024**
FA 24:1;O2	0.240	0.066	**−0.336**	**0.011**
LPC 20:5	−0.072	0.584	**−0.303**	**0.023**
LPC 20:4;O	−0.139	0.292	**−0.350**	**0.008**
PS O-32:0	−0.191	0.147	**−0.305**	**0.022**
PA O-30:2	−0.172	0.192	**−0.268**	**0.046**
LPC 18:4	−0.099	0.453	**−0.391**	**0.002**
Phosphorylcholine	−0.075	0.567	−0.183	0.177
ST 24:2;O4	0.008	0.949	**−0.281**	**0.036**

The bolded values are statistically significant.

**Table 4:** Correlations of lipid species with metabolic parameters in acromegaly group.

Lipid Species Label	Basal Glucose (mg/dL)		TG (mg/dL)		TC (mg/dL)		LDL-C (mg/dL)		HDL-C (mg/dL)	
	r	*p*	r	*p*	r	*p*	r	*p*	r	*p*
SM 34:0;O2	**0.280**	**0.035**	−0.090	0.501	−0.112	0.404	−0.196	0.147	0.126	0.353
PC 30:2	**0.287**	**0.030**	−0.082	0.536	−0.131	0.328	−0.162	0.232	−0.053	0.693
MGMG 18:2	**0.381**	**0.003**	−0.093	0.487	0.013	0.922	0.049	0.716	−0.116	0.393
Cer 36:0;O4	−0.005	0.688	**0.326**	**0.012**	−0.021	0.871	−0.170	0.207	−0.100	0.460
WE 34:1	0.110	0.413	0.112	0.403	−0.035	0.793	0.029	0.831	**−0.287**	**0.032**
LPS O-18:0	0.097	0.471	−0.001	0.991	0.152	0.256	0.004	0.975	**0.358**	**0.006**
DG 32:1	0.143	0.287	0.133	0.318	−0.072	0.589	0.028	0.832	**−0.357**	**0.006**
CE 15:0	0.017	0.896	0.049	0.711	−0.106	0.429	−0.070	0.608	**−0.285**	**0.033**
LPC 20:3	−0.022	0.866	0.044	0.742	−0.037	0.783	0.049	0.720	**−0.272**	**0.042**
WE 38:7	0.039	0.771	0.061	0.646	−0.064	0.628	−0.004	0.972	**−0.293**	**0.028**
LPS O-20:0;O	−0.031	0.815	0.124	0.355	0.200	0.133	**0.330**	**0.012**	−0.261	0.052
Cer 38:0;O4	−0.030	0.823	−0.063	0.637	**0.297**	**0.023**	0.255	0.057	0.189	0.164
ST 24:1;O3	0.130	0.334	0.130	0.330	**0.266**	**0.043**	0.184	0.174	−0.002	0.985

The bolded values are statistically significant.

## Data Availability

The original contributions presented in this study are included in the article/Supplementary Material. Further inquiries can be directed to the corresponding author.

## Supplementary Materials

The following supporting information can be downloaded at: https://www.mdpi.com/article/10.3390/ijms27021082/s1.

## Abbreviations

The following abbreviations are used in this manuscript:

AC	acromegaly group
ANOVA	one-way analysis of variance
AUC	area under the curve
BPC	base peak chromatograms
C	control group
CAR	carnitine
CE	cholesterol ester
Cer	ceramide
CerP	ceramide phosphate
CI	confidence interval
D	decrease
DG	diacylglycerol
FAs	fatty acids
FC	fold change
FDR	false discovery rate
FMF	find molecular feature
GH	growth hormone
HDL-C	HDL-cholesterol
HPLC-MS	high-performance liquid chromatography combined with mass spectrometry
HOMA-IR	homeostatic model assessment for insulin resistance
I	increase
LC-MS	liquid chromatography combined with mass spectrometry
LDL-C	LDL-cholesterol
LPA	lysophosphatidic acid
LPC	lysophosphatidylcholine
LPE	lysophosphatidylethanolamine
LPS	lysophosphatidylserine
LOOCV	leave-one-out cross-validation
MGMG	monogalactosyl monoacylglycerol
MRS	magnetic resonance spectroscopy
OGTT	oral glucose tolerance test
OR	odds ratio
PA	phosphatidic acid
PC	phosphatidylcholine
PCA	principal component analysis
PE	phosphatidylethanolamine
PLS-DA	partial least squares discriminant analysis
PS	phosphatidylserine
ROC	receiver operating characteristic
QC	quality control
SM	sphingomyelin
S/N	signal/noise
SPBP	sphingosine-1-phosphate
ST	sterol
TC	total cholesterol
TG	triglycerides
TIC	total ion chromatograms
ULN	upper limit of normal
VIP	variable importance in projection
WE	wax esters
