# Supplementary material for: Serum Lipidomic Profile Signature of Active Acromegaly and Relationships to Cardiovascular Disease
Source: Int J Mol Sci. 2026 Jan 21;27(2):1082. doi: 10.3390/ijms27021082 (PMC12841705; doi:10.3390/ijms27021082)
Supplement: Supplementary file 1 [file ijms-27-01082-s001.zip › Supplementary.pdf]

Table S1. Identification of 62 lipid species, including m/z values, species classification and IDs from Lipidmaps

m/z [M+1]	Putative Identification	Species short hand level/formula (Lipidmaps)	ID Lipidmaps or HMDB
**185.1127**	Phosphorylcholine	-	HMDB01565
**239.1602**	Hydroxy-tetradecadienoic acid	FA 14:3;O	LMFA01031188
**245.0768**	Hydroxy myristic acid	FA 14:0;O	LMFA01050001
**269.2074**	Heptadecenoic acid	FA 17:1	LMFA01030284
**301.1400**	Retinoic acid	$C_{20}H_{28}O_2$	LMPR01090019
**341.2689**	Docosanoic (behenic) acid	FA22:0	LMFA01010022
**377.3257**	Lithocholic acid	ST 24:1;O3	LMST04010003
**380.3354**	Sphingosine 1-phosphate	SPBP 18:1;O2	LMSP01050001
**391.2830**	6-Ketolithocholic acid	ST 24:2;O4	LMST04010146
**397.2938**	Hexadecyl-glycero-3-phosphate	LPA O-16:0	LMGP10060005
**399.3076**	Tetracosanedioic acid	FA 24:1;O2	LMFA01170039
**407.3349**	7-Ketodeoxycholic acid	ST 24:2;O5	LMST04010184
**414.3207**	Hydroxypalmitoleoylcarnitine	CAR 16:1;O	LMFA07070044
**419.2848**	Dihydroxycholesterol	ST 27:1;O3	LMST01010450
**421.3522**	Homodeoxycholic acid	ST 26:1;O4	LMST04020031
**424.3628**	Octadecadienoyl carnitine	CAR 18:2	LMFA07070009
**425.2136**	Octadecyl-glycero-3-phosphate	LPA O-18:0	LMGP10060004
**429.3164**	1,25-dihydroxyvitamin D2	$C_{28}H_{44}O_3$	LMST03010041
**455.3327**	Micromeric acid	$C_{30}H_{46}O_3$	LMPR0106180015
**458.3471**	O-carboxyheptadecanoyl)carnitine	CAR 18:1;O2	LMFA07070085
**468.3889**	tetradecanoyl-glycero-phosphocholine	LPC 14:0	LMGP01050012
**473.3441**	Chenodeoxycholic acid sulfate	ST 24:1;O4;S	LMST05020026
**482.4050**	Cer(d18:1/12:0)	Cer 30:1;O2	LMSP02010002
**484.3831**	O-behenoylcarnitine	CAR 22:0	LMFA07070089
**485.3459**	Lanoceric acid	FA 30:0;O2	LMFA01050534
**498.3990**	Hexadecanoyl-glycero-phosphoserine	LPS 16:0	LMGP03050002
**501.3260**	Palmitoleyl linolenate	WE 34:4	LMFA07010121
**502.3732**	Eicosatetraenoyl)-glycero-phosphoethanolamine	LPE 20:4	LMGP02050067
**507.3280**	Oleyl palmitate	WE 34:1	LMFA07010133
**512.4149**	LPS O-18:0	LPS O-18:0	LMGP03060002
**516.3877**	octadecatetraenoyl)-glycero-3-phosphocholine	LPC 18:4	LMGP01050040
**517.3317**	Octadecadienoyl-D-galactosyl-glycerol	MGMG 18:2	LMGL04010010
**526.4309**	octadecanoyl-glycero-phosphoserine	LPS 18:0	LMGP03050006
**531.3857**	Linolenyl stearate	WE 36:3	LMFA07010148
**542.4250**	Eicosapentaenoyl-glycero phosphocholine	LPC 20:5	LMGP01050050

**546.3989**	Eicosatrienoyl)-glycero-3-phosphocholine	LPC 20:3	LMGP01050133
**551.3535**	Retinyl oleate	WE 38:7	LMFA07011033
**556.4405**	LPS O-20:0;O	LPS O-20:0;O	LMGP03060018
**559.3215**	Arachidyl linolenate	WE 38:3	LMFA07010684
**560.4139**	Hydroxy-eicosatetraenoyl-glycero-phosphocholine	LPC 20:4;O	LMGP20010052
**567.3274**	Hexadecanoyl-hexadecenoyl)-glycerol	DG 32:1	LMGL02010010
**570.4565**	docosapentaenoyl)-glycero-3-phosphocholine	LPC 22:5	LMGP01050142
**575.4109**	DG(33:4)	DG 33:4	LMGL02010368
**590.4242**	CerP(d18:1/14:0)	CerP 32:1;O2	LMSP02050013
**591.3844**	DG (34:3)	DG 34:3	LMGL02010031
**595.3789**	DG(34:1)	DG 34:1	LMGL02010004
**600.4662**	Cer(t18:0/18:0)	Cer 36:0;O4	LMSP02030016
**604.4386**	PA O-30:2	PA O-30:2	LMGP10030004
**611.3524**	Cholesterol ester 15:0	CE 15:0	LMST01020027
**614.4818**	Cer(t18:0/19:0(2OH))	Cer 37:0;O4	LMSP02030065
**619.4364**	16:3 Cholesterol ester	CE 16:3	LMST01020039
**628.1931**	Cer(t18:0/20:0(2OH))	Cer 38:0;O4	LMSP02030017
**634.4492**	PE(28:1)	PE 28:1	LMGP02010407
**639.4043**	PA(32:5)	PA 32:5	LMGP10010061
**658.5069**	PE (30:3)	PE 30:3	LMGP02010367
**678.4742**	PC (28:0)	PC 28:0	LMGP01010390
**701.4156**	SM(d16:1/18:1)	SM 34:2;O2	LMSP03010040
**702.5318**	PC (30:2)	PC 30:2	LMGP01011323
**705.4869**	SM(d18:0/16:0)	SM 34:0;O2	LMSP03010004
**722.5010**	PS O-16:0/16:0	PS O-32:0	LMGP03020082
**776.2261**	PC O-18:0/18:0	PC O-36:0	LMGP01020091
**803.5372**	TG(16:0/16:1/16:1)	TG 48:2	LMGL03010018

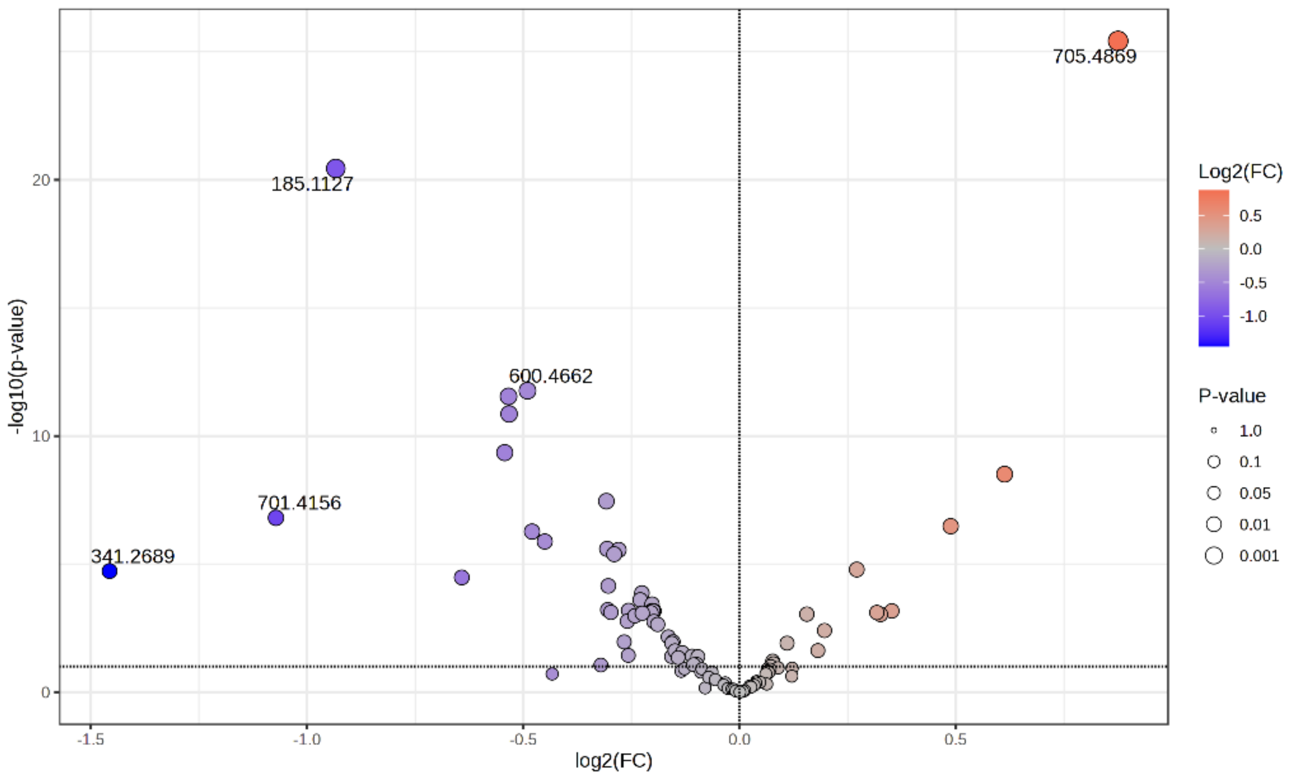

Figure S1. Volcano plot showing m/z values with increased MS intensities in the acromegaly group compared to the control group
